# Left ventricular strain changes at high altitude in rats: a cardiac magnetic resonance tissue tracking imaging study

**DOI:** 10.1186/s12872-024-03886-z

**Published:** 2024-04-24

**Authors:** Yanqiu Sun, Chenhong Zhang, Bo He, Lei Wang, Dengfeng Tian, Zhiqiang Kang, Lixin Chen, Ruiwen Li, Jialiang Ren, Yong Guo, Yonghai Zhang, Dingda Duojie, Qiang Zhang, Fabao Gao

**Affiliations:** 1 Department of Radiology, Qinghai Provincial People’s Hospital, Xining, China; 2 Department of Radiology, West China Hospital of Sichuan University, Chengdu, China; 3 Medical Equipment Management Office, Qinghai Provincial People’s Hospital, Xining, China; 4 Wuxi National Hi-tech Industrial Development Zone, GE Healthcare, 19 Changjiang Road, Wuxi, China; 5 Department of Radiology, People’s Hospital of Yushu Tibetan Autonomous Prefecture, Qinghai, China; 6 Department of Radiology, The Fifth People’s Hospital of Qinghai Province, Qinghai, China; 7 Department of neurosurgery, Qinghai Provincial People’s Hospital, Xining, China

**Keywords:** Plateau, Cardiac magnetic resonance, Tissue tracking, Ventricular function, Myocardial strain

## Abstract

**Background:**

Long-term exposure to a high altitude environment with low pressure and low oxygen could cause abnormalities in the structure and function of the heart. Myocardial strain is a sensitive indicator for assessing myocardial dysfunction, monitoring myocardial strain is of great significance for the early diagnosis and treatment of high altitude heart-related diseases. This study applies cardiac magnetic resonance tissue tracking technology (CMR-TT) to evaluate the changes in left ventricular myocardial function and structure in rats in high altitude environment.

**Methods:**

6-week-old male rats were randomized into plateau hypoxia rats (plateau group, *n* = 21) as the experimental group and plain rats (plain group, *n* = 10) as the control group. plateau group rats were transported from Chengdu (altitude: 360 m), a city in a plateau located in southwestern China, to the Qinghai-Tibet Plateau (altitude: 3850 m), Yushu, China, and then fed for 12 weeks there, while plain group rats were fed in Chengdu(altitude: 360 m), China. Using 7.0 T cardiac magnetic resonance (CMR) to evaluate the left ventricular ejection fraction (EF), end-diastolic volume (EDV), end-systolic volume (ESV) and stroke volume (SV), as well as myocardial strain parameters including the peak global longitudinal (GLS), radial (GRS), and circumferential strain (GCS). The rats were euthanized and a myocardial biopsy was obtained after the magnetic resonance imaging scan.

**Results:**

The plateau rats showed more lower left ventricular GLS and GRS (*P* < 0.05) than the plain rats. However, there was no statistically significant difference in left ventricular EDV, ESV, SV, EF and GCS compared to the plain rats (*P* > 0.05).

**Conclusions:**

After 12 weeks of exposure to high altitude low-pressure hypoxia environment, the left ventricular global strain was partially decreased and myocardium is damaged, while the whole heart ejection fraction was still preserved, the myocardial strain was more sensitive than the ejection fraction in monitoring cardiac function.

## Introduction

Over 140 million people live at high altitude (HA), that is, at greater than 2500 m above sea level [1]. The physiological response to low pressure / hypoxia varies with the altitude, our study site is at 3850 m of altitude. The decrease in air pressure in high altitude areas reduces the supply of oxygen [2]. Long-term exposure to a high altitude environment with low pressure and low oxygen can increase red blood cell levels, pulmonary artery pressure, cardiac afterload, and ultimately lead to changes in cardiac structure and function, and even cause high altitude heart disease (HAHD). HAHD is a common disease in the high altitude areas. HAHD primarily presents with right ventricular hypertrophy [3]. In severe cases, the left ventricle is also involved, and the cardiac dysfunction can ultimately lead to heart failure [4]. Interestingly, some studies have found that hypoxia can enhance myocardial resistance to ischemia and reperfusion in order to reduce myocardial injury induced by ischemia-reperfusion [5, 6]. NEW & NOTEWORTHY Chronic hypoxia and regular exercise are natural stimuli that confer sustainable myocardial protection against acute ischemia-reperfusion injury [7]. People at high altitudes seem to be more likely to avoid heart disease, of course, this depends on absolute altitude, genetic background and other factors [8]. Mice are genetically similar to humans to provide useful experimental data, especially in rats, which is the most important model of human cardiovascular disease. More importantly, rats are physiologically more like humans than mice. for example, the heart of a mouse beats at a rate of about 600 beats per minute, while the heart of rats beats at a rate less than two-thirds of the heart rate of mouse, closer to the average human resting rate of 70 beats per minute [9]. Therefore, we selected rats as the subject of study.

CMR-TT is a non-invasive, radiation-free, and contrast-free imaging technique that offers high stability and repeatability in evaluating the overall and local function of the left and right ventricles [10, 11]. CMR-TT has been successfully used to assess myocardial deformation in a Chinese cohort [12]. Ejection fraction as a traditional parameter has certain limitations, it cannot detect myocardial dysfunction early and reflect local left ventricular myocardial function accurately [13]. Patients with preserved ejection fraction in the early stage of cardiovascular disease may have obvious myocardial dysfunction. Myocardial strain is an emerging parameter that can evaluate the global and regional function of the heart [14].

In a previous study from our group [15], we found that the right ventricular structure and function changed in rats exposed to high altitude hypobaric hypoxic environment after 12 weeks, manifested as right ventricular end-diastolic volume, right ventricular end-systolic volume, right ventricular stroke volume, Right ventricular diastolic myocardial mass, Right ventricular systolic myocardial mass, tricuspid valve end systole caliber, right ventricular end-systolic Long axis and right ventricular end-diastolic long-axis levels were elevated. Pathological changes such as focal vacuolar degeneration and a small amount of inflammatory cell infiltration were seen under the microscope of the right ventricle. It is unknown whether the left ventricle also undergoes changes, we hypothesize that rats exposed to high-altitude and low oxygen environments for 12 weeks can cause changes in the structure, function, and pathology of the left ventricle.

In this study, we used CMR-TT to quantitatively evaluate the structural and functional changes in the left ventricular myocardium in rats exposed to a high altitude environment, the experimental data generated from this study will provide imaging evidence for the study of HAHD. Unlike our previous study, this study mainly explored the value of myocardial strain in assessing left ventricular function.

## Methods

### Animals

Six week-old male SPF Sprague Dawley (SD) rats (150.12 ± 22.23 g) were purchased from Chengdu Dossy Experimental Animals Co., Ltd. (Chengdu, China). The rats were randomly divided into two groups: the plateau group (*n* = 21) and the plain group (*n* = 10). The plateau rats were raised in the plateau animal laboratory (approximately 3,850 m; Yushu, Qinghai, China) for 12 weeks. The plain rats were maintained under a plain environment (approximately 360 m; Chengdu, Sichuan, China) for 12 weeks. The animals were housed in ventilated cages at 18–26 °C and 45–70% humidity under natural light conditions. The animals had free access to food (SPF grade) and water. The disposal of animals during the experiment complied with the Pain Management.

Standards in the Eighth Edition of “Guidelines for the Care and Use of Laboratory Animals” [16]. Experiments were performed under a project license [No. SYXK (Qing) 2019-0001] granted by the ethics committee of Qinghai Provincial Department of Science and Technology, in compliance with the Drug Inspection and Testing in Qinghai Province institutional guidelines for the care and use of animals. A protocol was prepared before the study without registration.

### CMR-TT data acquisition

All scans were performed on a 7.0T Bruker Biospec 70/30 MRI scanner (Bruker, Germany). The scanner was equipped with radiofrequency birdcage coils of 72-mm inner diameter (I.D.) and surface array receiving coils of 56-mm I.D. The rat was placed in a sealed glass chamber and anesthetized with premixed 2–3% isoflurane in oxygen on a MATRX VIP 3000 small animal anesthesia machine (Midmark Co., USA). The rat’s chest was positioned in the center of the coil. ECG electrodes were inserted into each forelimb and the right hindlimb. A breath sensor was placed on the abdomen. After the breath and ECG signals stabilized, images were acquired successively at three orientations (coronal, sagittal and axial plane) in the region of the chest and used to localize the heart. After that, a cardiac four chamber view cine SSFP sequence was performed to localize the left ventricular short axis. Cine-Flash-flc imaging data were subsequently acquired in the short axis plane from the apex to the base of the heart. The imaging parameters were: echo time 2.5 ms, repetition time 8 ms, flip angle 20°, slice thickness 1.5 mm, gap between adjacent slices 0 mm, field of view 50 mm × 50 mm, matrix size 256 × 256, number of excitations 4. Fifteen frames of 8–10 slices each were acquired per cardiac cycle. The images acquired included the short and long axis views of the left ventricles and the two- and four-chamber hearts.

### CMR-TT data analysis

**Fig. 1:**
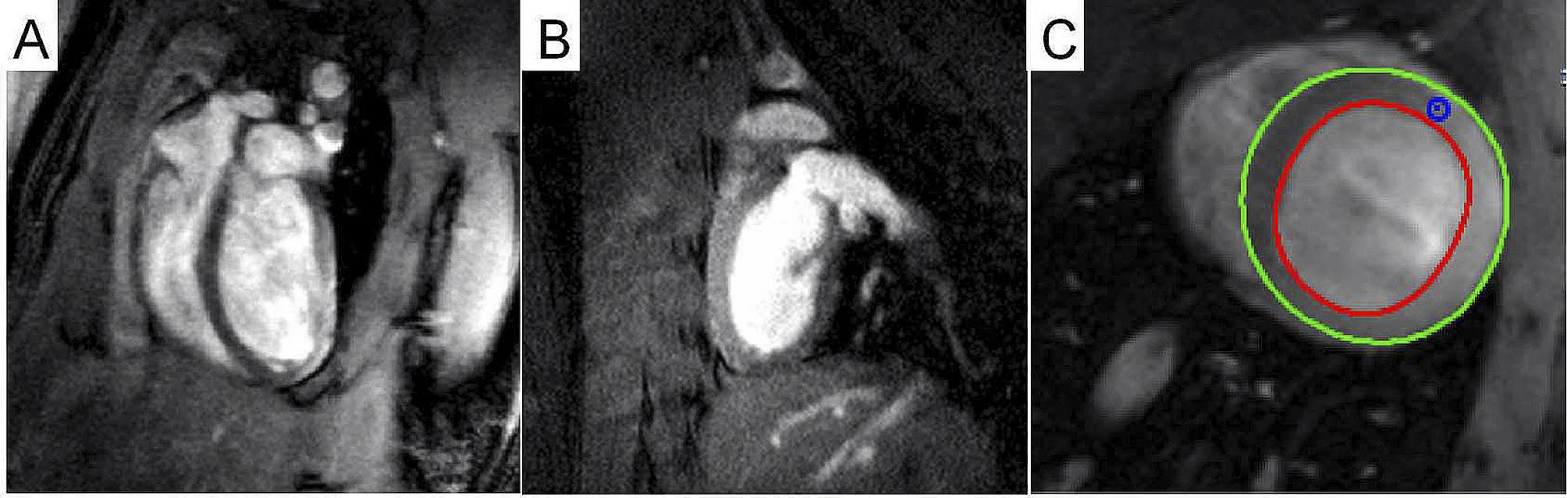
Typical CMR cine images. (**A**) Four-chamber view. (**B**) Two-chamber left view. (**C**) left ventricular endo- and epicardial borders at end-diastole

**Fig. 2:**
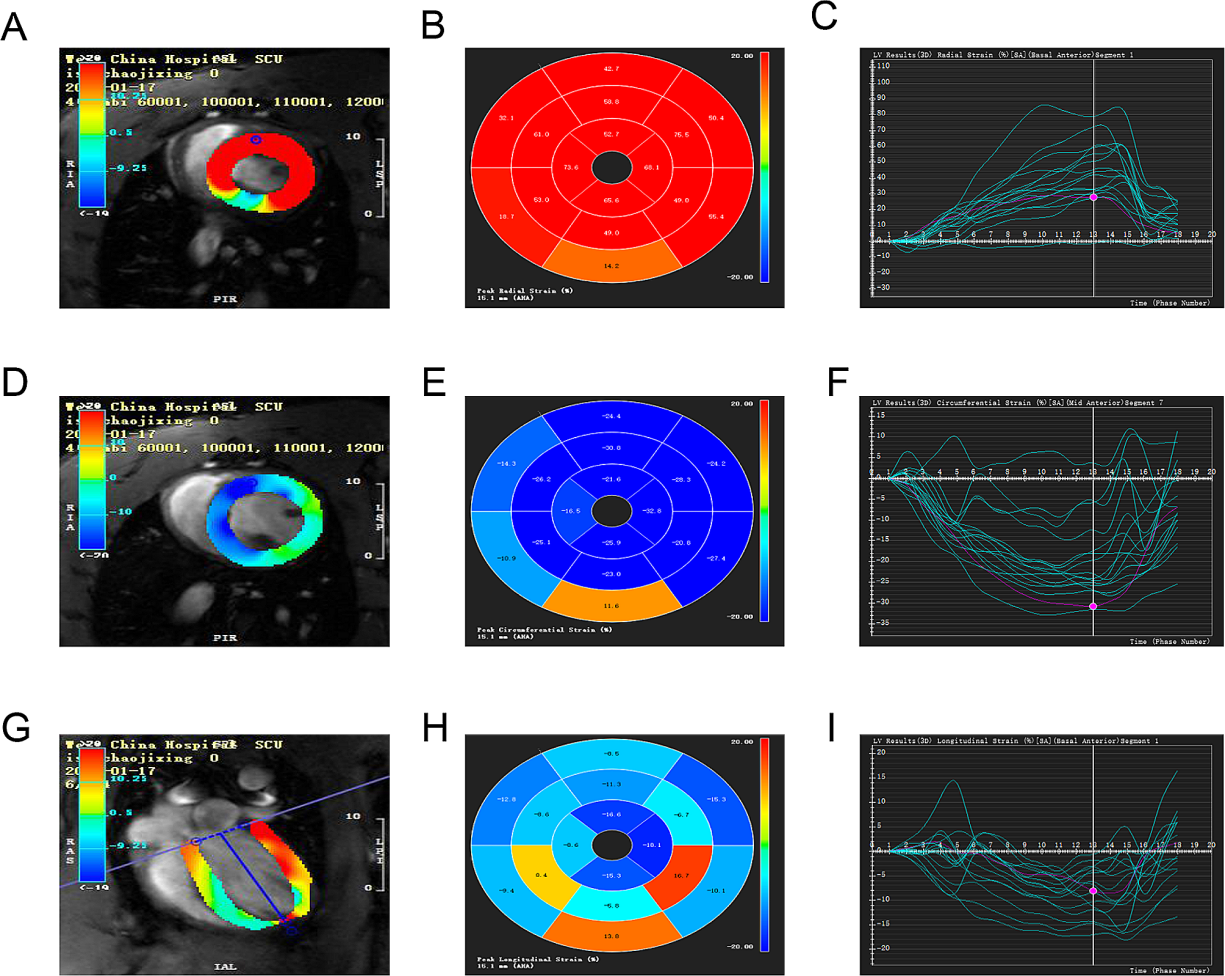
3D analysis of the left ventricular myocardial strain. (**A, D, G**) Endocardial border determination for GRS (**A**), GCS (**D**), and GLS (**G**) analysis. (**B, E, H**) 16-segment bull’s eye map of GRS (**B**), GCS (**E**), and GLS (**H**). (**C, F, I**) GRS (**C**), GCS (**F**), and GLS (**I**) curves

The CMR-TT imaging data were processed using the cvi42 software (Circle Cardiovascular Imaging Inc., Canada) by a radiologist with substantial CMR experience. The endo- and epicardial borders at end-systole and end-diastole were carefully drawn out. The papillary muscles were included while the trabecula was excluded (Fig. 1). The software automatically tracks on-screen pixels during the cardiac cycle. The cvi42 SD short model was used to calculate the left ventricle functional parameters including the end-diastolic volume (EDV), end-systolic volume (ESV), stroke volume (SV), and ejection fraction (EF). We added continuous short axis and 3 long axis images to the Tissue Tracing module for strain analysis. At the end of diastole, the left ventricular epicardium was delineated and tissue tracking was performed. The software automatically obtained 3D results, and the 3D strain was obtained from both the short axis and long axis directions. This study included the following strain parameters: 3D global circumferential strain (GCS), 3D global longitudinal strain (GLS), and 3D global radial strain (GRS). (Fig. 2).

### Histopathology

After CMR-TT data acquisition was completed, the rats were sacrificed by injection of potassium chloride via the tail vein. The hearts were harvested, cleaned, fixed in 10% formaldehyde, embedded in paraffin, and sectioned. For histopathological evaluation, the tissues were dewaxed, stained with hematoxylin-eosin (HE), and subjected to microscopic examination.

### Statistical analysis

The SPSS software (version 23.0) was used for data interpretation. All results are presented as mean ± standard deviation (SD). Data were verified for normality and compared using the independent *t* test. *P* value less than 0.05 was deemed statistically significant.

## Results

### Left ventricular function

**Fig. 3:**
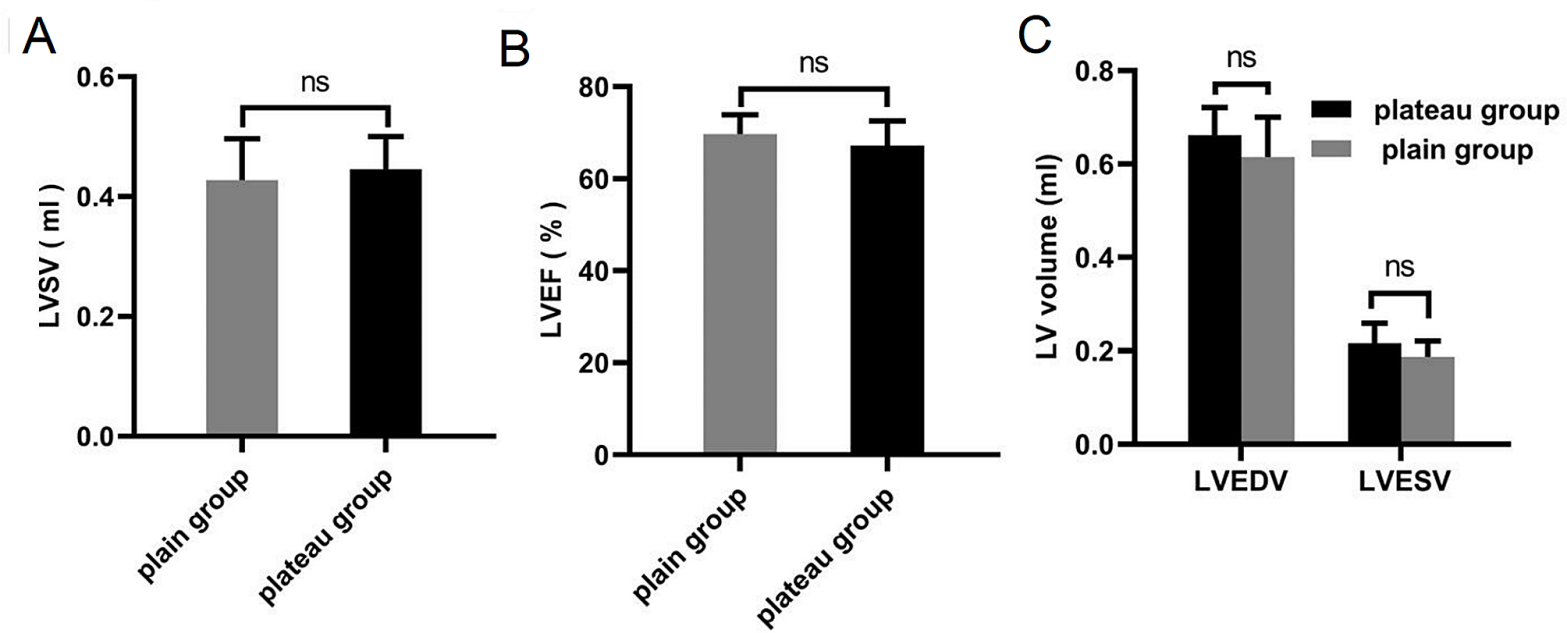
Left ventricular functions of the plain and plateau rats. No significant difference in left ventricular SV, EF, EDV or ESV was detected between the two groups (*P* > 0.05, Fig. **A-C**). plain: *n* = 10, plateau: *n* = 21, ns: not statistically significant

In this study, we determined the left ventricular EDV, ESV, SV, and EF based on the CMR cine imaging data. The typical CMR cine images are shown in Fig. 1. The average left ventricular EDV, ESV, SV and EF were calculated to be 0.66 mL, 0.22 mL, 0.44 mL and 67.12%, respectively, for the plateau rats and 0.62 mL, 0.19 mL, 0.43 mL and 69.12%, respectively, for the plain rats. No significant difference in left ventricular EDV, ESV, SV or EF was detected between the two groups (*P* > 0.05, Fig. 3A-C; Table 1).

**Table 1:** Comparison of left ventricular cardiac function parameters between two groups of rats

Index	plateau group(*n* = 21)	plain group (*n* = 10)	t	*P*
LVEDV/mL	0.66 ± 0.06	0.62 ± 0.09	1.750	0.091
LVESV/mL	0.22 ± 0.04	0.19 ± 0.03	1.969	0.059
LVSV/mL	0.44 ± 0.05	0.43 ± 0.07	0.716	0.480
LVEF/%	67.12 ± 5.32	69.69 ± 4.18	-1.335	0.192
LVGCS/%	-19.28 ± 4.39	-22.11 ± 2.35	1.899	0.068
LVGLS/%	-13.58 ± 3.51	-18.12 ± 3.52	3.367	0.002
LVGRS/%	36.82 ± 11.36	45.32 ± 8.43	-2.099	0.045

### Left ventricular myocardial strain

**Fig. 4:**
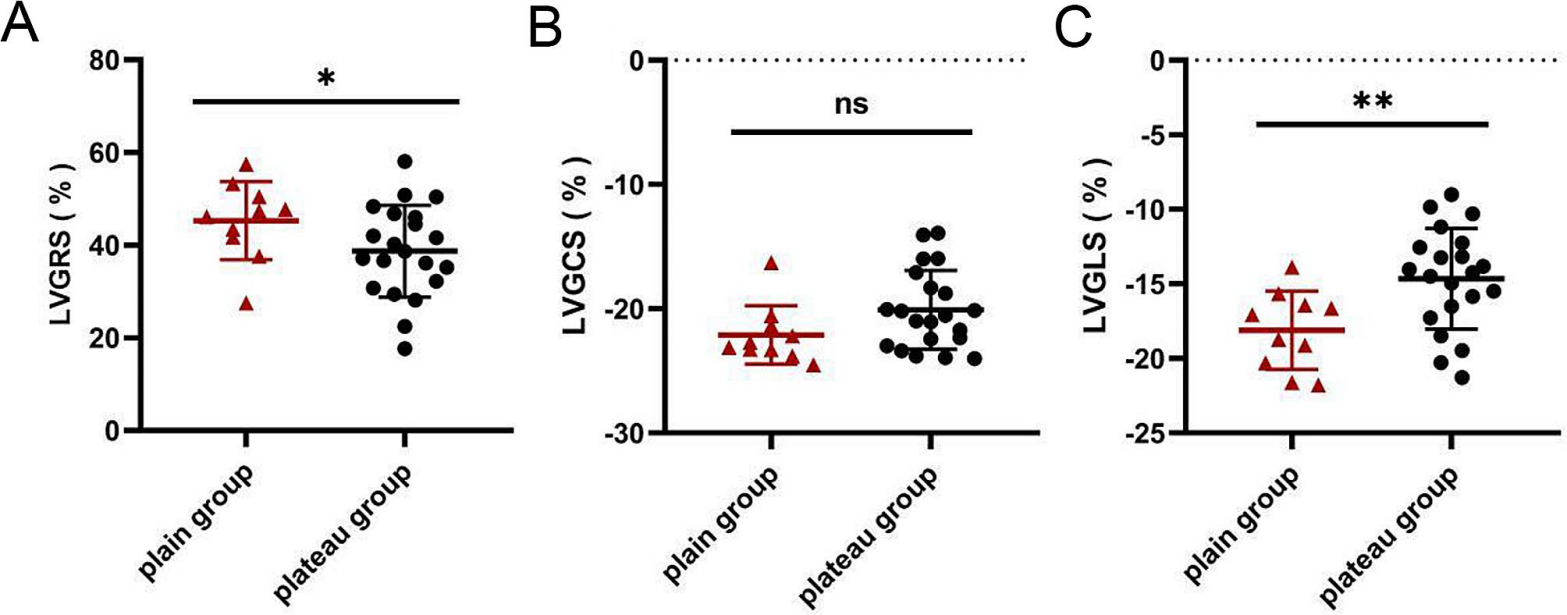
Left ventricular myocardial strain parameters of the plain and plateau rats. (**A−C**) Left ventricular GRS (**A**), GCS (**B**), and GLS (**C**). plain: *n* = 10, plateau: *n* = 21, ns: not statistically significant, *P < 0.05, **P < 0.01

In this study, we determined the left ventricular strain parameters including GRS, GCS and GLS based on the CMR-TT imaging data. 3D analysis of the left ventricular myocardial strain is illustrated in Fig. 2. The left ventricular strain parameters are presented in Fig. 4; Table 1. Compared with the plain rats, the plateau rats showed significantly lower left ventricular GRS (36.82% vs. 45.32%, *P* < 0.05, Fig. 4A) and GLS (-13.58% vs. -18.12%, *P* < 0.05, Fig. 4C). No significant difference in the left ventricular GCS was detected between the two groups (*P*>0.05, Fig. 4B).

### Myocardial histopathology results

**Fig. 5:**
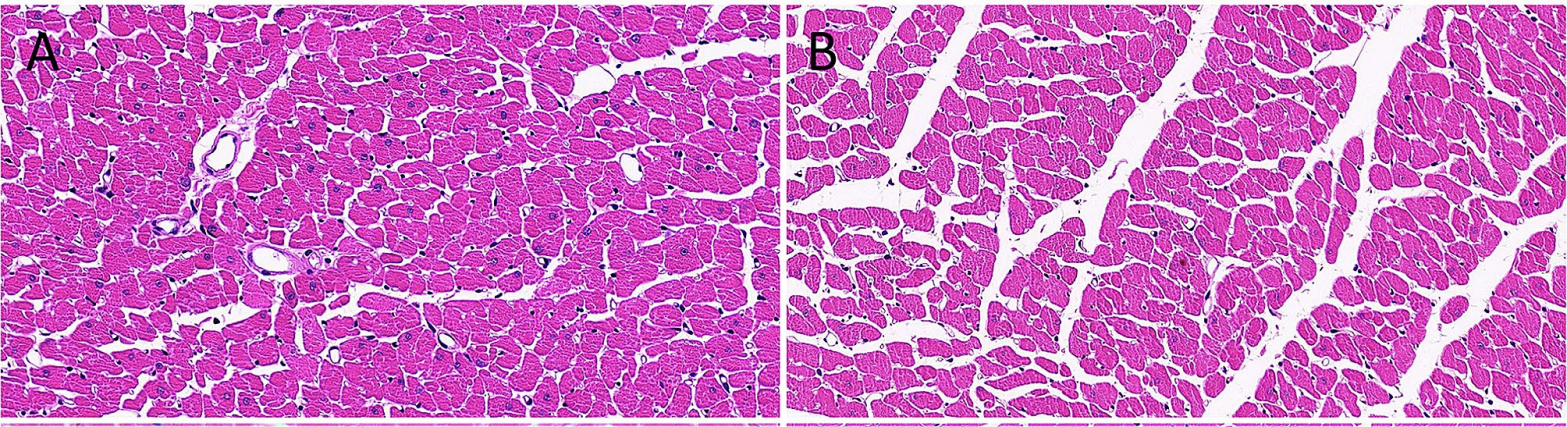
HE of left ventricle in plain group and plateau group. (**A**) The normal myocardial fibers of left ventricle in plain group (objective: ×40) under high magnification. (**B**) The normal myocardial fibers of left ventricle in plateau group (objective: ×40) under high magnification, respectively

HE staining was used for histological examination of the ventricular myocardium. Under microscopy, both the left ventricular myocardium of the plain rats and left ventricular myocardium of the plateau rats appeared normal, showing well-organized muscle strips, and morphologically healthy cardiomyocytes with a clearly defined nucleus (Fig. 5A, B).

## Discussion

High altitude heart disease (HAHD) was a chronic high-altitude disease, which referred to the right ventricular hypertrophy caused by persistent pulmonary hypertension and myocardial cell hypoxia after people in plain areas migrated to the low-pressure and hypoxic environment at high altitude, and finally the right ventricular dysfunction caused by decompensation, in the late stage, serious cardiovascular system dysfunction such as left ventricular hypertrophy and expansion and total heart failure can also occurred [16]. The course of high altitude heart disease was slow, and the incidence increased gradually with the extension of living time at high altitude. HAHD had many pathogenesis mechanisms, and the fundamental reason was that the stress reactions regulated the cardiovascular system through nerves, body fluids, endocrine systems, etc., resulting in the increase of heart rate, pulmonary arterial pressure, and red blood cells(RBC), improving the oxygen carrying capacity of red blood cells to meet tissue oxygen supply [17–19]. Hypoxia could induce the synthesis of erythropoietin (EPO) mediated by hypoxia inducible factor (HIF), thus stimulating the production of red blood cells and increasing the levels of hemoglobin (HGB) and hematocrit (HCT ) [20]. The increase of red blood cells helped to improve the oxygen carrying and transport capacity in blood. However, the excessive increase of HGB and HCT levels would also increase blood viscosity and the additional burden on the heart, which would eventually reduce cardiac output and aggravate hypoxia, and further develop into HAHD [16]. Hypoxic pulmonary hypertension and thickening of pulmonary arterioles were the key link and fundamental feature of the pathogenesis of HAHD. Long-term hypoxia stimulation could lead to pulmonary artery hyperplasia and pulmonary vascular remodeling [21, 22]. Pulmonary vascular remodeling could cause an increase in pulmonary artery pressure and right ventricular afterload, resulting in right ventricular hypertrophy and dysfunction, and ultimately left heart dysfunction and failure. Of note, testosterone had also been reported to increase RBC, HGB and HCT levels by stimulating EPO [23], while estrogen appeared to have the opposite effects [24]. In this study, we included male SD rats as the research object, in addition to hypoxia stimulation, testosterone may also participate in and improve the blood oxygen transport capacity of rats at high altitude, which would be interesting to find out whether female plateau rats have similar changes in oxygen transport capacity in future studies.

In a previous study from our group found that RVEDV, RVESV and RVSV of rats were significantly increased after continuous exposure to hypoxia for 12 weeks, while RVEF remained normal, and the levels of RBC, HGB and HCT in blood of rats were significantly increased [15], indicating that chronic hypoxia at high altitude had changed the structure and function of the right ventricle, in order to adapt to hypoxia at high altitude, the pumping function of the right ventricle increased compensably, and the myocardial contractility remained unchanged. However, due to the increase of cardiac output and afterload, the overall strain of the myocardium decreased.

It had been reported that myocardial strain was an important predictor of cardiovascular disease [25, 26]. Previous studies on high altitude heart disease mainly focused on the right heart, while few studies on the changes of myocardial stress in the left heart caused by high altitude hypoxia. Therefore, we used 7T CMR-TT technology to explore the effects of high altitude hypoxia on left ventricular function and myocardial strain in rats. The results showed that LVGRS and LVGLS decreased, while LVEDV, LVESV, LVSV, LVGCS and EF remained at normal levels, which showed that the left ventricular strain of rats had changed, and the left ventricular myocardial injury occurred after continuous exposure to high altitude for 12 weeks. However, in order to meet the tissue oxygen supply, the left ventricular systolic function was still preserved. There was inconsistency between EF and myocardial strain in the evaluation of cardiac function. A study performed a combined mathematical and echocardiographic study to understand the inconsistencies between EF and strains, which had shown that increased wall thickness and/or reduced EDV augment EF, and therefore could maintain a normal EF despite reduced shortening. EF was quadratically dependent on circumferential shortening and only linearly dependent on longitudinal shortening; hence, EF was less sensitive to a reduction in longitudinal shortening, this study also suggested that strain measurements reflect systolic function better than EF in patients with preserved EF [27].

In this study, we also found that LVGRS and LVGLS decreased differently, and LVGLS decreased more significantly, indicating that LVGLS was more sensitive to assess early cardiac function injury than LVGRS and LVGCS, which may be related to the different arrangement of myocardial bundles. Torrent Guasp et al. [28] found that the characteristics of left ventricular myocardial contraction were related to the arrangement of myocardium. There were two kinds of arrangement of left ventricular myocardial bundles, namely, the longitudinal myocardial bundle forming the endocardium and epicardium and the circumferential myocardial bundle forming the middle layer of the myocardium. The longitudinal myocardial bundle runs from the basal segment to the apical segment, bypasses the apex and returns to the basal segment, forming a spiral structure [29]. In addition, the degree and direction of myocardial deformation were different due to the different directions of endocardial and epicardial myocardial fibers. The contraction of endocardial fibers leaded to the longitudinal shortening of the myocardium, and the contraction of epicardial fibers leaded to the circumferential shortening of the myocardium, which could make the myocardial radial increase [30]. Duo to the coronary artery had its own unique shape, the farthest blood supply area was the endocardium, when myocardial hypoxia occurred, the endocardial myocardium was first affected, followed by the epicardial myocardium, so the reduction of longitudinal strain was more significant [30].

### Advantages and limitations

Based on the true replication of high altitude hypobaric and hypoxic environment, we observed the changes of cardiac function of rats under hypobaric and hypoxic environment. This study provided experimental and theoretical basis for the future study of high altitude heart disease. However, this study still has some limitations. Firstly, due to the lack of 7.0T MRI scanning equipment in the plateau area, after being raised in a high altitude environment for 12 weeks, we transported plateau rats to the plain area for CMR scanning and data acquisition, which takes about 14 h and may have an impact on our experimental data. Secondly, we did not assess pulmonary artery pressure, arterial partial pressure of oxygen and / or oxygen saturation, nor did we observe the relationship between pulmonary artery pressure and changes in right ventricular function, which will be our next research plan.

## Conclusions

In summary, after continuous exposure to high altitude hypoxia for 12 weeks, the left ventricular myocardium of rats was damaged, while global contractility was still preserved. Global longitudinal strain (GLS) had shown superior performance in detecting cardiac dysfunction caused by high altitude hypoxia, GLS preferably reflected cardiac systolic function with preserved EF, which may be a useful tool for early diagnosis of HAHD.

## Data Availability

All data generated or analysed during this study are included in this article.

## Abbreviations

CMR-TT: Cardiac magnetic resonance tissue tracking
CMR: Cardiac magnetic resonance SD, Sprague Dawley
LV: Left ventricularEF, ejection fraction
EDV: End-diastolic volume
ESV: End-systolic volume
SV: Stroke volume
GLS: Global longitudinal strain
GCS: Global circumferential strain
GRS: Global radial strain
HE: Hematoxylin-eosin
HA: High altitude
HAHD: High altitude heart disease
RBC: Red blood cells
EPO: Erythropoietin
HIF: Hypoxia inducible factor
HGB: Hemoglobin
HCT: Hematocrit
